# Osteomyelitis of the Patella in a 10-Year-Old Girl: A Case Report and Review of the Literature

**DOI:** 10.1155/2017/6573271

**Published:** 2017-04-18

**Authors:** Matthias Sperl, Michael Novak, Daniela Sperl, Martin Svehlik, Georg Singer, Tanja Kraus

**Affiliations:** ^1^Department of Pediatric Orthopedics, Medical University of Graz, Graz, Austria; ^2^Division of Pediatric Hemato-Oncology, Medical University of Graz, Graz, Austria; ^3^Department of Pediatric and Adolescent Surgery, Medical University of Graz, Graz, Austria

## Abstract

The incidence of osteomyelitis constantly declines. While the disease most commonly affects the long bones, involvement of the patella is rarely seen. Due to this rarity and the variable clinical presentation, diagnosis is often delayed. The present case report describes a 10-year-old female patient with a delayed diagnosis of patella osteomyelitis. The diagnostic procedures and the treatment regimen are described. Additionally, a detailed literature review of the available publications reporting osteomyelitis of the patella in children is presented.

## 1. Introduction

In western industrialized countries, acute osteomyelitis represents a rare disease in children. Nevertheless, its severe sequelae such as growth disturbances, early joint destruction, and the resulting lifelong disability are well known and feared. While acute osteomyelitis of the long bones is more common, involvement of the patella is seldom encountered [1, 2]. In 1829, Thirion described the first case of acute patella osteomyelitis [3]. Due to its variable clinical presentation, diagnosis is frequently delayed.

We report the case of an otherwise healthy 10-year-old girl with osteomyelitis of the left patella. The patient's history and the management of the case are presented. Additionally, a review of the currently available literature reporting osteomyelitis of the patella is given.

## 2. Case Report

A 10-year-old girl complained about pain of the left knee after an accident sustained during trampoline jumping. The patient had a superficial skin abrasion but due to decreasing pain no doctor was consulted. Three weeks later she walked 35 kilometres in three days during a school trip causing moderate pain of her left knee mainly during night-time. However, the pain was not associated with swelling, redness, or fever. The family consulted a medical practitioner, a pediatrician, and an orthopaedic surgeon. Radiographs of the left knee were performed and no alterations of the patella were described. The patient was treated with NSAID.

**Figure 1:**
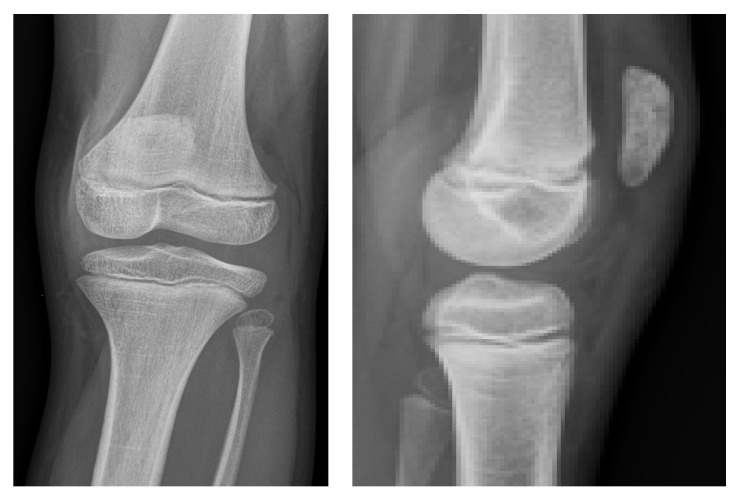
Radiographs of the knee joint 8 weeks after the first symptoms. A hypodense area is visible at the upper pole of the patella.

**Figure 2:**
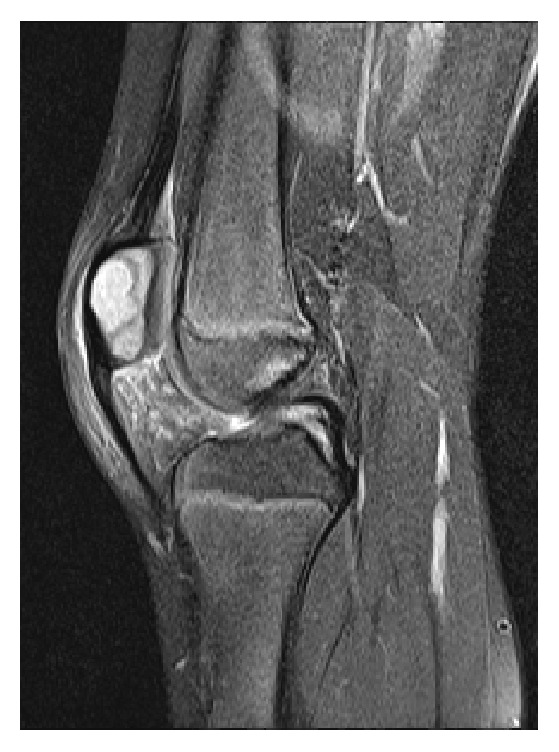
MRI showing a sharply limited enhancement of the patella. The radiologist suspected a chondroblastoma.

Due to persisting pain the local hospital was consulted eight weeks after the first symptoms. Physical examination did not reveal any abnormalities except a painful leg extension. The erythrocyte sedimentation rate was 19 mm/h (normal range: 1–10 mm/h) and the antistreptolysin-titer was 352 IU/ml (normal value: −200 IU/ml). For further diagnostics, another radiograph and a subsequent MRI of the left knee were performed showing an osteolytic lesion with a diameter of 1.5 cm of the patella with central necrosis (Figures 1 and 2). Radiologists and an orthopaedic surgeon interpreted the lesion as a chondroblastoma of the patella and a follow-up with MRI was planned within 3 months.

**Figure 3:**
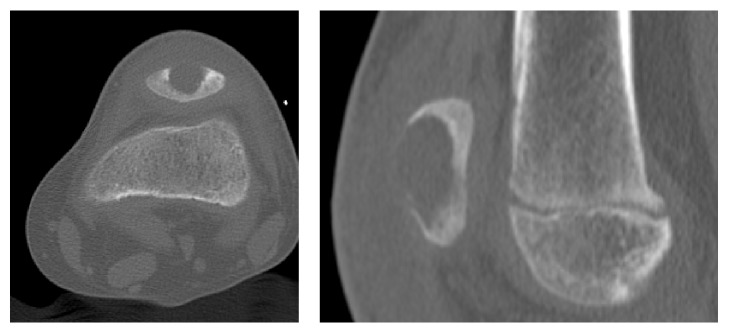
CT of the left knee clearly showing an osteolytic process with sharp borders.

Five days later the girl returned because of increasing pain of her left knee. Clinical inspection showed swelling and hyperthermia of the left knee. The white blood cell count was 8.750/*μ*l (normal values 5.50–13.50/*μ*l), the erythrocyte sedimentation rate was 61 mm/h (normal range 1–10 mm/h), and the C-reactive protein (CRP) was 9.3 mg/dl (normal range 0.0–1.1 mg/dl). A CT scan revealed a 2.0 × 1.1 × 1.0 cm osteolytic lesion of the patella associated with a destruction of the ventral corticalis (Figure 3).

The girl was now referred to our Pediatric Orthopedic Centre. The girl presented with slight swelling of the prepatellar soft-tissue and local hyperthermia of her left knee without joint effusion or fever. Biopsy and a swap were performed in general anaesthesia using a ventral approach. Histological examination showed signs of acute osteomyelitis. Therefore, debridement and curettage were indicated. Intraoperatively, the bone defect measured 1 × 1.5 × 1 cm. A gentamicin chain (Septopal Minikette, Pharmalog Pharma Logistik GmbH, Boennen, Germany) was placed into the defect and removed after seven days. The histological examination of the curettage verified acute osteomyelitis and* Staphylococcus aureus* was grown from the culture. Additionally, the patient was treated with intravenous cefuroxime (100 mg/kg bodyweight in three doses) and fosfomycin (190 mg/kg bodyweight in three doses) for 12 days followed by 16 days of oral antibiotics using amoxicillin and clavulanic acid (37 mg/kg bodyweight in three doses). The left leg was immobilized in a long leg cast. Following a hospital stay of 17 days the girl was discharged free of pain and the immobilization was continued for another two weeks.

Two weeks later the patient was seen in the outpatient department. She was free of pain and the clinical examination of the knee was unremarkable. CRP and the white blood cell count were within normal ranges. The cast was removed and the patient was sent home without restrictions for daily life activities.

**Figure 4:**
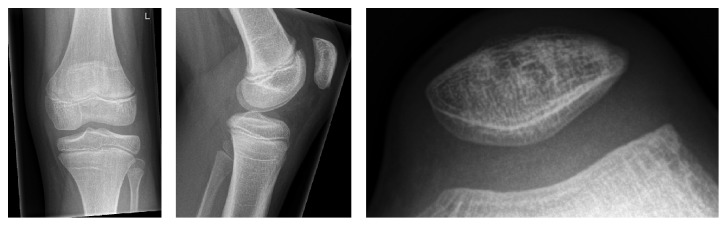
Follow-up radiographs 17 months after surgery revealing an almost normally appearing patella.

At the latest follow-up examination after 17 months the girl was symptom-free with full range of knee joint motion. Radiographs showed a nearly full osseous recovery of the patella (Figure 4).

## 3. Discussion

The incidence of osteomyelitis during growth is described with 2.9 per 100,000 children with declining values in industrialized countries [16]. A peak of incidence has been found in children aged between 5 and 15 years [1, 7, 9, 10, 13, 17, 18]. In this age group, osteomyelitis is most commonly caused by haematogenous spread of an infection with an occult source [2, 9, 19]. While most cases of osteomyelitis are diagnosed in the long bones, involvement of the patella is rarely diagnosed [2, 19]. The patella possesses a rich anastomotic arterial network consisting of contributions from the superior and inferior geniculates and the anterior tibial recurrent arteries [18]. The high number of immune cells in such an extensive vascular network might explain the rare occurrence of haematogenous osteomyelitis of the patella [1, 9, 18]. Additionally, the patella does not have a physeal plate and the associated sluggish hemodynamics have also been considered to explain the rarity of this disease in this location [1, 9].

The correct diagnosis of osteomyelitis of the patella is often delayed with initially suspected diagnoses including septic arthritis, synovitis, septic bursitis, and peripatellar cellulitis [7]. Other differential diagnoses of patellar lesions include Sinding-Larsen-Johansson disease, bipartite patella, and a variety of rare tumors such as chondroblastomas, giant cell tumors, osteoid osteomas, and aneurysmal bone cysts but also malignant tumors [20]. In the patient described in the present case report chondroblastoma was primarily suspected on both conventional radiographs and MRI. The underlying reasons for the late diagnosis are the rarity and the variable presentation of the disease. In children with osteomyelitis of the patella, knee pain can range from mild to severe eventually associated with limping and/or restriction of joint motion. Moreover, leucocyte count and plane radiographs can be inconspicuous early in the course of the disease [1, 9, 13, 17]. Due to these variabilities, bone scintigraphy, CT, and gadolinium enhanced MRI have been recommended as diagnostic modalities [9, 17]. Even though growth disturbances due to patellar osteomyelitis are uncommon, spreading to other parts of the skeleton or local intrusion into the knee joint with subsequent purulent arthritis represents a feared complication. Therefore, prompt diagnosis and therapy are still demanded. To ensure this, a high index of suspicion, close clinical monitoring, and early advanced imaging are highly recommended in children with persistent peripatellar swelling, pain, hyperthermia, and particularly fever and/or elevated inflammatory parameters.

Our review of the literature revealed that reports describing pediatric osteomyelitis of the patella are confined to case reports or very small case series. In total we were able to analyze 14 reports including 27 children. Detailed information about treatment and cultures of these patients is presented in Table 1. Even though there are cases with negative bacterial cultures,* Staphylococcus aureus,* like in the present case report, seems to represent the most commonly found infecting organism [2, 19]. Treatment with antibiotics is undisputed and may even be enough for uncomplicated cases of patellar osteomyelitis [19]. However, the duration and the preferred routes of administration, that is, oral or parenteral, greatly vary between the different reports (see Table 1). In accordance with Dartnell et al., surgical treatment consisting of debridement and curettage represents the recommended treatment for absent improvement despite antibiotic treatment and complicated patients like the girl described in the present report [1, 19]. In patients without joint involvement, arthrotomy does not seem to be necessary. However, no consensus exists regarding the use of antibiotic chains. Advantages of the application of antibiotic chains include the possibility of delivering high local doses of antibiotics without the nephrotoxic and ototoxic side effects [21]. The great disadvantage, however, is the necessity of a second operation for chain removal. Nevertheless, our treatment regimen consisting of parenteral and subsequent oral antibiotic treatment combined with local gentamicin therapy via a chain led to a favourable outcome despite late diagnosis.

In conclusion, correct diagnosis of pediatric patellar osteomyelitis is frequently delayed. In order to ensure prompt and correct diagnosis, an exact clinical examination, laboratory tests, plain radiographs, and in equivocal cases MRI are indispensable.

## Figures and Tables

**Table 1:** Literature review including information about treatment and cultures of pediatric patients with osteomyelitis of the patella.

Author	Cases	Treatment	Culture
Gil-Albarova et al., 2012 [2]	1 girl	Debridement and curettage; 10 days of i.v. cefotaxime/cloxacillin → 2 weeks of oral cloxacillin	*Staph. aureus*

De Gheldere, 2009 [4]	1 boy	Joint and patella aspiration; 10 days of i.v. rifampicin and cloxacillin → 6 weeks of rifampicin and clindamycin orally	*Staph. aureus*

Choi, 2007 [1]	2 boys	Curettage and i.v. cefazedone followed by oral antibiotics	Case I: negative Case II: *Staph. aureus*

Masuda et al., 1999 [5]	1 boy	Curettage; one week of cefuroxadine → cefmetazole and cefuroxadine for 2 months	Negative

Moyikoua et al., 1993 [6]	1 boy	Gentamicin for 3 weeks	N/A

Roy et al., 1991 [7]	2 girls 2 boys	Case I: antibiotics → incision and drainage Case II: antibiotics → arthroscopy and incision of the patella Case III: incision, debridement, and drainage Case IV: incision, debridement	3x *Staph. aureus* 1x *Clostridium bifermentans*

Papavasiliou and Sferopoulos, 1989 [8]	1 girl 2 boys	Curettage and lavage, parenteral antibiotic treatment	*Staph. aureus* in all cases

Cahill, 1978 [9]	2 boys	Case I: antibiotics → curettage and parenteral antibiotics Case II: initial treatment with antibiotics, exploration of the knee and inflow-outflow cefalotin irrigation tubes, and cefalotin for another 5 days.	Case I: gram positive cocci Case II: negative

Vaninbroukx et al., 1976 [10]	1 girl 2 boys	Case I: sequestrectomy, arthrotomy, and parenteral antibiotics Case II: initial treatment with antibiotics → sequestrectomy and parenteral antibiotics Case III: antibiotics → curettage	Case I: N/A Case II: negative Case III: N/A

Kochhar and Srivastava, 1976 [11]	1 child (4 adults)	Antibiotic treatment in all cases, three patients underwent patellectomy	*Staph. aureus* in all cases

Wadlington et al., 1971 [12]	1 boy	Antibiotics: penicillin, methicillin, colistin, and gentamicin	*Pseudomonas aeruginosa*

Angella, 1967 [13]	1 girl 1 boy	Case I: initial treatment with antibiotics → curettage, parenteral antibiotics Case II: 28 days of parenteral antibiotics, aspiration of the knee joint two times	Case I: *Staph. aureus* Case II: negative

Evans, 1962 [14]	2 girls 2 boys (1 adult)	Case I: antibiotics Case II: antibiotics (knee aspiration) Case III: removal of a sequestrum Case IV: penicillin for 4 weeks, removal of a sequestrum	Case I: none Case II: *Staph. aureus* Case III: none Case IV: *Staph. aureus*

Schafer, 1952 [15]	1 boy	Initial treatment with antibiotics → arthrotomy → antibiotics	*Staphylococcus*
